# Optogenetic perturbation of lipid droplet localization affects lipid metabolism and development in *Drosophila*

**DOI:** 10.1016/j.jlr.2025.100848

**Published:** 2025-06-20

**Authors:** Xin Deng, Wei Wang, Dandan Peng, Luhao Zhang, Zhihao Ma, Junfen Fu, Chao Tong, Yingke Xu

**Affiliations:** 1 Department of Endocrinology, Children's Hospital of Zhejiang University School of Medicine, National Clinical Research Center for Child Health, Hangzhou, China; 2 MOE Key Laboratory of Biomedical Engineering, State Key Laboratory of Extreme Photonics and Instrumentation, Department of Biomedical Engineering, Zhejiang University, Hangzhou, China; 3 MOE Key Laboratory for Biosystems Homeostasis & Protection and Innovation Center for Cell Signaling Network, Life Sciences Institute, Zhejiang University, Hangzhou, China; 4 Innovation Center for Smart Medical Technologies and Devices, Binjiang Institute of Zhejiang University, Hangzhou, China

**Keywords:** lipid droplet, optogenetics, *Drosophila*, oogenesis, metabolism, development

## Abstract

Lipid droplets (LDs) are dynamic organelles crucial for lipid storage and homeostasis. Despite extensive documentation of their importance, the causal relationship between LD localization and function in health and disease remains inadequately understood. Here, we developed optogenetics-based tools, termed “Opto-LDs,” which facilitate the interaction between LDs and motor proteins in a light-dependent manner, enabling precise control of LD localization within cells. Utilizing these optogenetic modules, we demonstrated that light-induced relocation of LDs to the periphery of hepatocytes results in elevated very-low-density lipoprotein (VLDL) secretion, recapturing the beneficial effect of insulin in vitro. Furthermore, our studies in transgenic *Drosophila* revealed that proper LD localization is critical for embryonic development, with mistargeting of LDs significantly affecting egg hatching success. In summary, our work underscores the great importance of LD localization in lipid metabolism and development, and our developed tools offer valuable insights into the functions of LDs in health and disease.

Lipid droplets (LDs) are cytosolic organelles that sequester neutral lipids and are conserved across species from bacteria to humans (1). Unlike other organelles with bilayer membranes, LDs have a unique monolayer phospholipid structure essential for their formation, growth, and shrinkage (2). They act as energy reservoirs, providing a steady supply of lipids regardless of external nutrient availability. Additionally, LDs are increasingly recognized as key regulators of lipid metabolism, orchestrating lipid absorption, distribution, storage, and metabolism within the cell. They also play a role in signaling cascades and can be precursors to other molecules such as hormones and lipoproteins (3). Studies have shown that LDs are implicated in cellular stress, potentially due to their role in sequestering excess lipids and toxic proteins (4). LDs navigate along microtubules or actin filaments using motor proteins, altering their cellular distribution through clustering and dispersion. This motility may facilitate organelle interactions, reorganization during different metabolic states, and dysregulation in diseases (5).

Given the myriad biological roles of LDs in other cells, it appears likely that they also have critical functions during *Drosophila* oogenesis. They are highly motile in nurse cells and oocytes, moving in linear paths and switching directions every few seconds (6, 7). However, the underlying mechanisms and functions of LDs motility for oogenesis and embryo development are not well understood. Current technology enables the real-time tracking of LDs within cells and the identification of the organelles with which they interact (8). For instance, treatment of COS-7 cells with nocodazole disrupts the microtubule network, inhibits LD movement, and diminishes interactions between LDs, mitochondria, and peroxisomes. Furthermore, LD motility increases following viral immune stimulation, leading to LD accumulation and altered LD–mitochondrial interactions, which underpin metabolic changes in the cell (9). However, the precise relationship between LD motility and lipid metabolism is not yet fully understood, as most studies have explored this relationship indirectly. Other factors, such as nutrition and signaling pathways, may confound the results.

Conventional methods for perturbing organelle positioning typically involve genetic or pharmacological approaches that modulate motor proteins and the dynamics of the cytoskeleton, including microtubules and actin filaments (10). These treatments often severely impair normal cell function as they broadly affect cellular cargo transportation. In contrast, optogenetics offers a novel and precise tool for manipulating specific cellular processes using light, including cellular signaling, protein function, organelle transport, and interactions (11, 12, 13, 14, 15). Optogenetics has significant advantages over conventional techniques for controlling organelle distribution, such as high spatiotemporal accuracy, minimal interference with other cellular functions, and dynamic activation at single-cell or even subcellular resolution. Previous studies have utilized light-induced protein heterodimerization systems, including cryptochromes (CRYs) (16), light-oxygen-voltage (LOV)-domain (16, 17), and phytochrome B (PhyB) (18), to control organelle positioning, providing the approach for studying the causal roles of organelle transport and distribution in cellular functions.

In this study, we combined optogenetics and live-cell microscopy to control LD movement (Opto-LDs) and to investigate how perturbations in LD localization impact lipid metabolism and animal development. Utilizing the blue light-responsive CRY2 and its binding partner CIBN, we engineered the association of LDs with distinct motor proteins, directing their movement toward the cell periphery (plus ends of microtubule) or perinuclear regions (minus ends of microtubule). In hepatocytes, we found that the spatial localization of LDs correlates with cellular metabolic status and is dependent upon an intact microtubule network. Our further studies indicated that light-induced peripheral accumulation of LDs enhances the secretion of very low-density lipoprotein (VLDL), consistent with the effect of insulin in increasing TG secretion in primary hepatocytes. Extending our investigation to *Drosophila*, we used a germline-specific nanos-Gal4 driver to express Opto-LDs. Confocal imaging of ovaries demonstrated the global redistribution of LDs after blue light stimulation. The treatments significantly reduced the hatching rate of the embryos without affecting egg-laying of the flies, revealing that proper LD localization is essential for *Drosophila* development. Our developed Opto-LDs system, which allows for minimally invasive and reversible control of LD distribution, has facilitated the understanding of the link between LD distribution, lipid metabolism, and animal development. We anticipate that this tool will have vast applications in lipid biology.

## Materials and methods

### Materials

The BODIPY493/503 (#D3922), BODIPY558/568 C12 (#D3835), Hoechst 33,342 (#62249), and Lipofectamine 3000 Transfection Reagent (#L3000008) were acquired from Invitrogen (Thermo Fisher Scientific). Nocodazole (NOC; #M1404-2 MG) was obtained from Merck Millipore (Merck). The mCherry antibody (#ab167453) was purchased from Abcam. All other reagents were obtained from Sigma unless otherwise noted.

The KIF5A–EGFP-CIBN (Addgene plasmid, #102252), BICDN-CIBN (Addgene plasmid, #102253), and mCherry-CRY2clust (Addgene plasmid, #105624) plasmids were kindly provided by Prof. Liting Duan (The Chinese University of Hong Kong). The PLIN2(1–191)–mCherry-CRY2, EMTB-EGFP, and EMTB-miRFP647nano3 plasmids were synthesized by GENEWIZ (China). To generate the control construct, the CRY2 sequence was removed from the PLIN2(1–191)–mCherry-CRY2 plasmid. BICDN-EGFP-CIBN was constructed through overlap PCR (polymerase chain reaction) by inserting the EGFP gene into the backbone of BICDN-CIBN. The transgenic vectors constructed for *Drosophila* strains were based on the pUASp-attB vector (DGRC Stock 1358; https://dgrc.bio.indiana.edu//stock/1358; RRID: DGRC_1358). The pUASp-attB-Lsd-1-mCherry-CRY2^P2A^-KIF5A-CIBN plasmid was obtained by inserting the Lsd-1-mCherry-CRY2^P2A^-KIF5A-CIBN sequence into the pUASp-attB vector. The control plasmid, pUASp-attB-Lsd-1-mCherry^P2A^-KIF5A-CIBN, was similarly generated. The expression of the inserted DNA fragments in adult female germline cells was driven by a nanos-Gal4 driver (TB00145) from the Tsinghua Fly Center.

### Cell culture and transfection

The COS-7 and LO2 cells used in this study were purchased from the American Type Culture Collection (ATCC). COS-7 and LO2 cells were cultured in Dulbecco's Modified Eagle Medium (DMEM), supplemented with 10% fetal bovine serum (FBS) and penicillin-streptomycin (Beyotime), at 37°C in a humidified atmosphere containing 5% CO_2_. Transfection was performed using Lipofectamine 3000 (Life Technologies) Transfection Reagent according to the manufacturer's protocol.

### Fly strains

*Drosophila* strains were cultivated on standard medium at 25°C with 60% humidity, under a 12-h light: 12-h dark photoperiod. Transgenic strains were created using microinjection techniques. All transgenic lines were produced by PhiC31-mediated integration, which facilitates the targeted insertion of DNA fragments into specific genomic loci (the landing site 25C6 of attp40 was used), resulting in presumed single-copy transgenes. Optogenetic *Drosophila* strains were generated by crossing *UASp-Lsd-1-mCherry*^*P2A*^*-KIF5A-CIBN* (Control) *or UASp-Lsd-D1-mCherry-CRY2*^*P2A*^*-KIF5A-CIBN* (Opto-LDs) flies with *nanos-Gal4* flies. Individual virgin females of the genotypes *UASp-Lsd-1-mCherry*^*P2A*^*-KIF5A-CIBN; nanos-Gal4* (Control) or *UASp-Lsd-1-mCherry-CRY2*^*P2A*^*-KIF5A-CIBN; nanos-Gal4* (Opto-LDs) were mated with single *w1118* male flies in vials to promote ovarian development at 25°C with 60% humidity under either a 24-h blue light (488 nm, 2.2 mW/cm^2^) or a 24-h dark photoperiod (see in Fig. 4B).

**Fig. 1:**
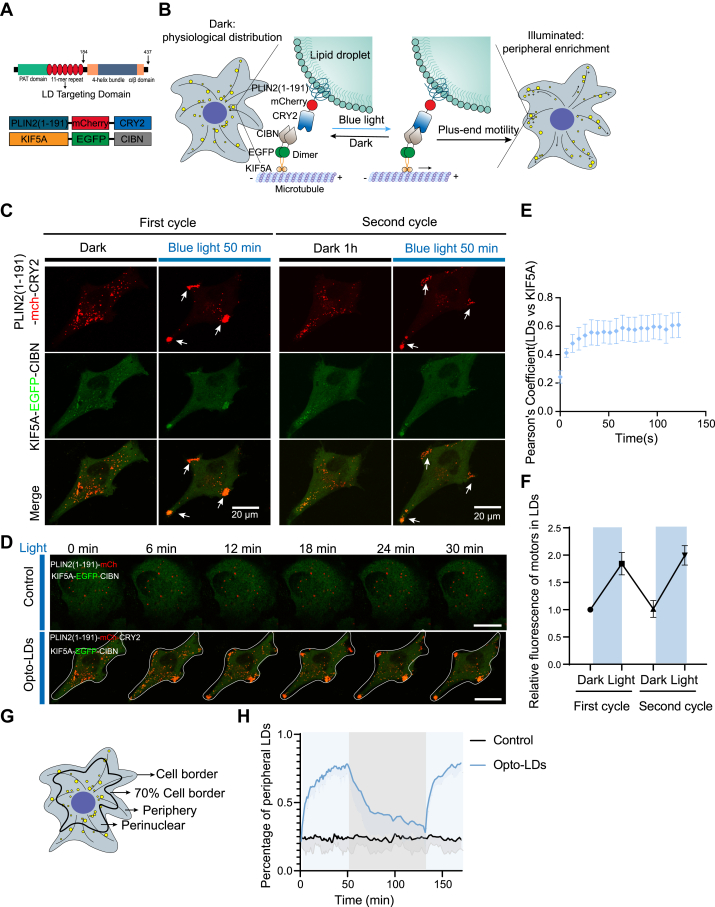
**Light-induced distribution of LDs to cell periphery**. A: schematic representation of the predicted domain architecture of human PLIN2, with amino acid numbers indicated, and the diagrams of the generated PLIN2(1–191)-mCherry-CRY2 and KIF5A-EGFP-CIBN constructs. Note: The PLIN2(1–191) sequence contains a highly conserved PAT domain and an 11-mer repeat region, predicted to form amphipathic helices, which facilitate the targeting of co-expressed proteins to the LD surface. B. Schematic depiction of optogenetics-induced redistribution of LDs to the cell periphery. LDs (yellow) are tagged with PLIN2(1–191) fused to CRY2. CIBN is co-expressed with a truncated kinesin, KIF5A. Blue light exposure induces the interaction of CIBN and CRY2, recruiting kinesin motors to the LDs and driving their transport toward the microtubule plus end, thereby promoting peripheral localization. C-H: Live-cell imaging (C–D) and quantification (E–H) of COS-7 cells expressing PLIN2(1–191)-mCherry-CRY2 and KIF5A-EGFP-CIBN before and after 50 min of blue light illumination (5 mW 488 nm laser; 60 s interval). Cells underwent 2 cycles of blue light illumination (50 min each), followed by 60 min of recovery in the dark (see also Video S1). LDs were moving toward the cell periphery after blue light stimulation, as indicated by white arrows. D: Time-lapse images of LDs movement. The top panel showed COS-7 cells transfected with PLIN2(1–191)-mCherry and KIF5A-EGFP-CIBN (Control) and the bottom panel showed the Opto-LDs transfected cells under light simulation. E: Colocalization analysis of PLIN2(1–191)-mCherry-CRY2 (LDs) and KIF5A-EGFP-CIBN (KIF5A) over time (n = 10). F: The relative fluorescence of KIF5A-EGFP-CIBN (motors) upon LDs in both dark and light conditions under two cycles of stimulus. G: The cell periphery was defined as 70% distance from the cellular outline and the remaining fraction was defined as the perinuclear region. H: Graphs illustrating the percentage of cell periphery LDs in both Control and Opto-LDs transfected cells under either dark or light stimulated conditions. Data are represented as mean ± SEM from 10 cells for Opto-LDs and 8 cells for the Control group analyzed across 3 independent experiments. Scale bars, 20 μm.

**Fig. 2:**
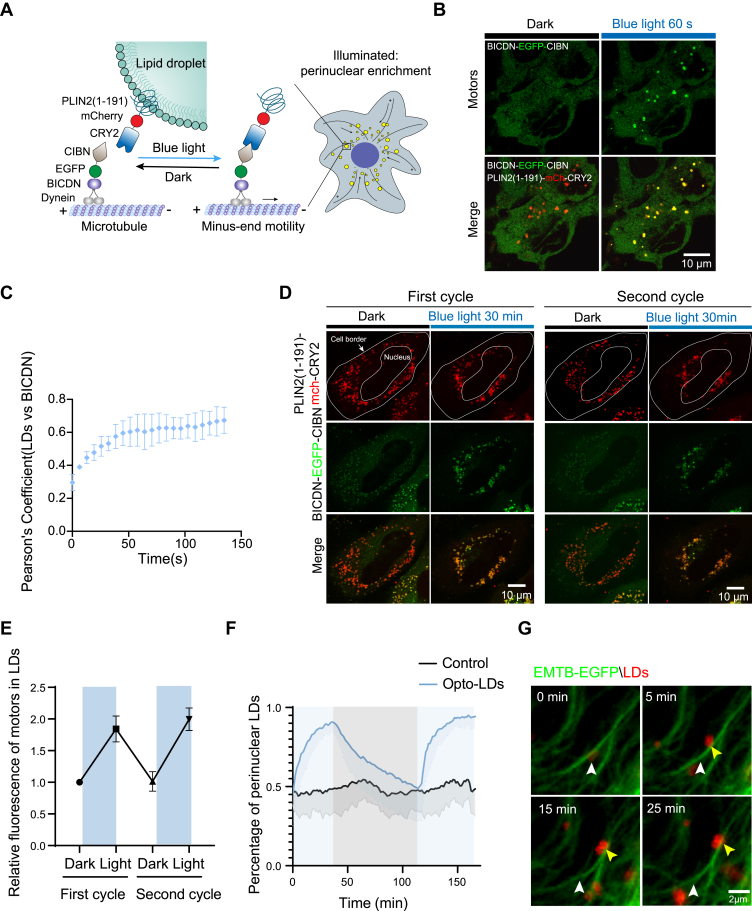
**Light-induced perinuclear localization of LDs.** A: Schematic depiction of the optogenetics-induced perinuclear movement of LDs. LDs (yellow) are tagged with PLIN2(1–191) fused to CRY2. CIBN is linked to BICDN, a dynein/dynactin adaptor protein. Blue light illumination promotes heterodimerization between CIBN and CRY2, recruiting dynein to LDs and directing their movement toward the microtubule minus end, promoting perinuclear transport. B-C: Live-cell imaging (B) and colocalization analysis (C) of PLIN2(1–191)-mCherry-CRY2 (LDs) and BICDN-EGFP-CIBN (BICDN) over time. (B) The top panel showed the motors before and after 60 s of blue light stimulation. The bottom panel showed the merged images of motors and LDs in both dark and light simulated conditions. D-G: Live-cell imaging (D) and quantification (E–F) of COS-7 cells expressing PLIN2(1–191)-mCherry-CRY2 and BICDN-EGFP-CIBN, before and after blue light illumination (5 mW 488 nm laser; 60 s interval). Cells underwent 2 cycles of blue light illumination (30 min each), followed by 60 min of recovery in the dark (see also Video S2). E: The relative fluorescence of BICDN-EGFP-CIBN (motors) in LDs in both dark and light conditions under two cycles of stimulus. F: Graphs illustrating the percentage of cell perinuclear LDs in both Control and Opto-LDs transfected cells in either dark or light stimulated conditions. Data are represented as mean ± SEM from 13 cells for Opto-LDs and 8 cells for Control group analyzed across 3 independent experiments. G: LD movement towards the cell perinuclear region, indicated by yellow arrows, and the starting position marked by white arrows, occurs via light-induced recruitment of dynein along microtubules. Cells were co-transfected with BICDN-CIBN and PLIN2(1–191)–mCherry–CRY2, along with EMTB-EGFP, utilized to visualize microtubules.

**Fig. 3:**
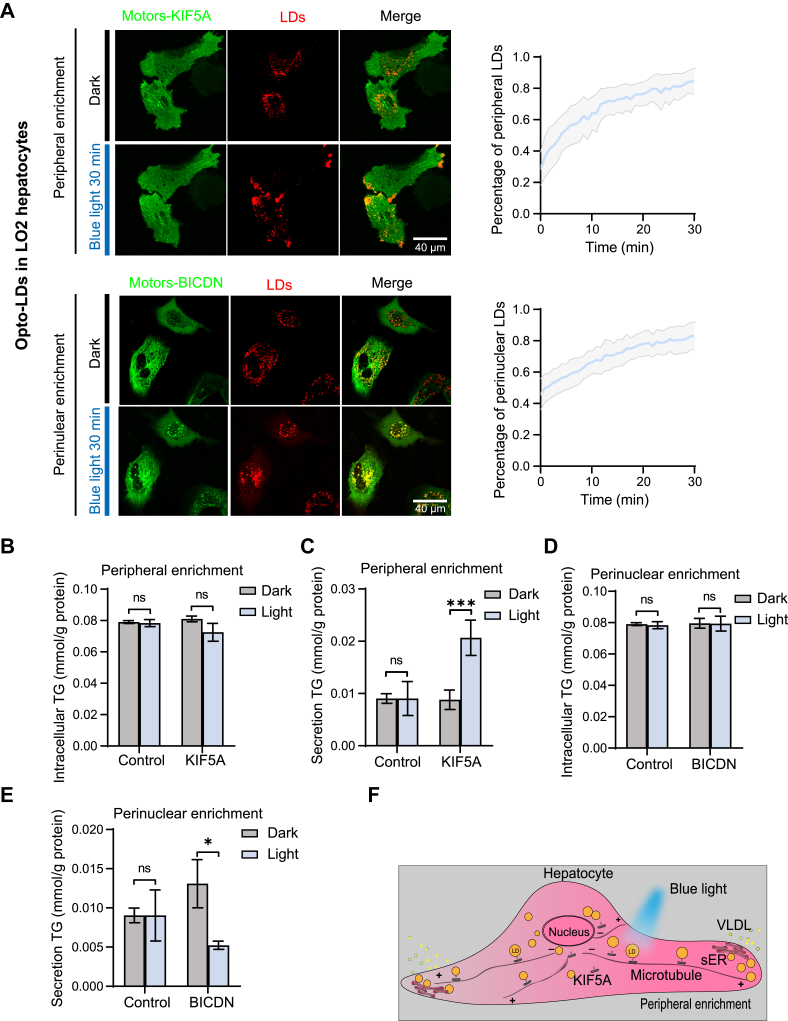
**Localization of LDs affects lipid metabolism in hepatocytes**. A: Live-cell confocal imaging and quantification of Opto-LDs in LO2 hepatocytes transfected with PLIN2(1–191)-mCherry-CRY2 and either KIF5A-EGFP-CIBN (for peripheral enrichment) or BICDN-EGFP-CIBN (for perinuclear enrichment) before and after blue light stimulation for 30 min. B-C: Quantification of both intracellular (B) and secreted (C) triglyceride (TG) levels in LO2 cells transfected with PLIN2(1–191)-mCherry-CRY2 and KIF5A-EGFP-CIBN (Opto-LDs) or with PLIN2(1–191)-mCherry and KIF5A-EGFP-CIBN (Control group). Cells were subjected to pulsed blue light LED array illumination (1 min on, 1 min off, 2 mW/cm^2^) for 16 h. D–E: Quantification of both intracellular (D) and secreted (E) triglyceride (TG) levels in LO2 cells transfected with PLIN2(1–191)-mCherry-CRY2 and BICDN-EGFP-CIBN (Opto-LDs) or with PLIN2(1–191)-mCherry and BICDN-EGFP-CIBN (Control group). Cells were subjected to pulsed blue light LED array illumination (1 min on, 1 min off, 2 mW/cm^2^) for 16 h. F: Schematic representation of light-induced LD transport to the periphery in hepatocytes. Data are represented as mean ± SEM. ∗*P* < 0.05; ∗∗*P* < 0.01; ∗∗∗*P* < 0.001 by *t* test. Scale bars, 10 μm.

**Fig. 4:**
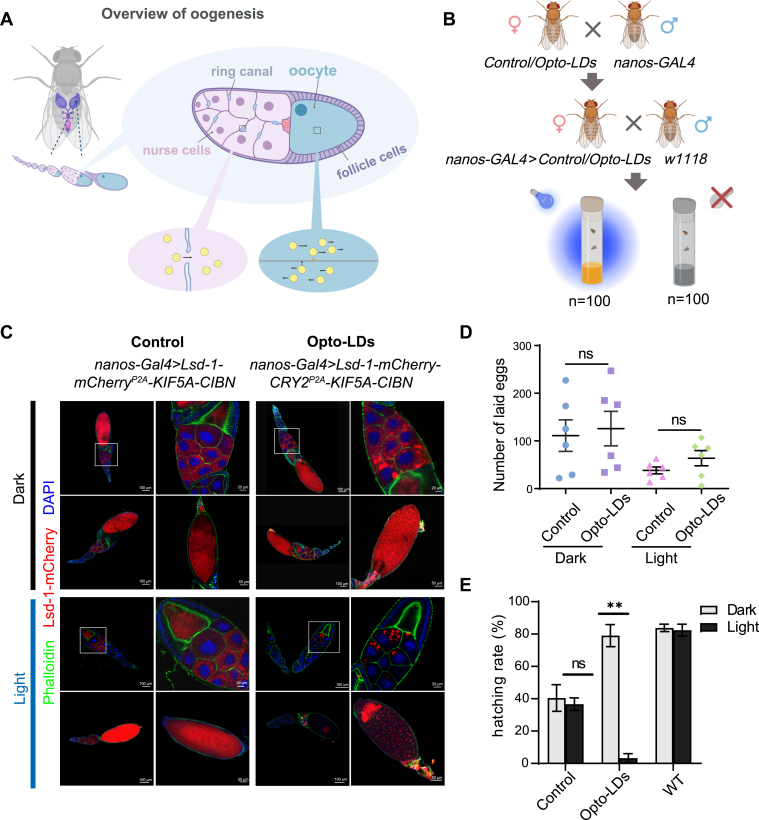
**Sustained light-induced relocation of LDs in *Drosophila* oocyte**. A: Overview of *Drosophila* oogenesis and LDs motility. Left: A schematic illustration of *Drosophila* oogenesis in one ovariole. Right: a representative mid-stage egg chamber. Oogenesis consists of 14 stages of follicle development. Each follicle consists of a layer of somatic epithelial cells, termed follicle cells, surrounding sixteen germline-derived cells; fifteen of these are support cells, termed nurse cells, and the other is the oocyte. Nurse cells supply the growing oocyte with nutrients, organelles, and signaling molecules. During *Drosophila* mid-oogenesis, many LDs are generated in the nurse cells. Bottom left: cytoplasmic streaming transports LDs from nurse cells through ring canals into the oocyte. Bottom right: In the oocyte cytoplasm, most LDs move passively by cytoplasmic streaming; a subset is actively transported by motors along microtubules. B: Schematic representation of the experimental treatment in *Drosophila*, where *UASp-Lsd-1-mCherry*^*P2A*^*-KIF5A-CIBN* (Control) or *UASp-Lsd-1-mCherry-CRY2*^*P2A*^*-KIF5A-CIBN* (Opto-LDs) transgenic flies were crossed with *nanos-Gal4* flies and kept in a sustained 24-h blue light (488 nm, 2.2 mW/cm^2^) or a 24-h dark photoperiod. The resulting virgin females carrying both *Gal4* and *UAS* transgenes were then mated with *w1118* male flies and subjected to either a sustained 24-h blue light (488 nm, 2.2 mW/cm^2^) or a 24-h dark photoperiod. C: Confocal imaging of ovarioles from adult females expressing *nanos-Gal4*> *Lsd-1-mCherry*^*P2A*^*-KIF5A-CIBN* (Control) or *nanos-Gal4*> *Lsd-1-mCherry-CRY2*^*P2A*^*-KIF5A-CIBN* (Opto-LDs) flies in the dark and blue light stimulated conditions. Ovaries were stained with phalloidin-Alexa Fluor 488 and DAPI, with genotypes indicated above the images. mCherry signal indicating Lsd-1 labeled LDs. Scale bars, 100 μm, 50 μm or 20 μm as labeled. D: Quantification of the number of laid eggs in 1.5 h from crosses of 60 females and 20 males with indicated genotypes in the dark or after light stimulation. E: The hatching rate of eggs from Control and Opto-LDs expressing female flies crossed with *w1118* male flies, subjected to sustained 24 h blue light or 24 h dark photoperiod. 30 eggs were examined in independent experiments (n = 7). Data are presented as mean ± SEM.

### Fertility measurements

To ensure successful mating, females and *w1118* males were cohabited for a minimum of 5 days. Then the flies were transferred to a new food tube, and the eggs were examined after 2 days. The emerged flies were counted 14 days later. Each cross was treated as an independent biological replicate, consisting of a single pair of female and male flies, with 100 replicates conducted per experimental condition. All experiments were conducted under either 24-h blue light or 24-h dark conditions. The proportion of tubes with eggs indicated egg-laying capacity (Fig. 4D), while the proportion of tubes with emerged flies indicated fertility (Fig. 4E).

### Egg-laying and hatching experiments

For the egg-laying experiments, newly emerged virgin flies cohabited with *w1118* male flies (60 females and 20 males per cross) in a bottle for 4 days. On the fifth day, the flies were transferred into a cage with a plate containing normal food at the bottom. After a 2-h adaptation period, the plate was replaced with a fresh food plate, and the flies were allowed to lay eggs for 1.5 h. The eggs on the plate were then counted. Six independent biological replicates were conducted for each treatment. For the hatching experiments, newly laid eggs were transferred to a fresh food plate, with 30 eggs per plate and 7 replicates per experimental group. Seventy-two hours after egg-laying, emerging larvae were counted, and the hatching rate was calculated as the ratio of larvae to eggs.

All experiments were conducted under either 24-h blue light or 24-h dark conditions. Fly or embryo transfers for the dark groups were conducted under red light.

### Immunofluorescence staining

Ovaries from mated adult female flies were dissected on the third day post-emergence and fixed in 4% paraformaldehyde (PFA; Sigma-Aldrich, #158127) for 30 min at room temperature (RT). After three washes with 1× PBS, the ovaries were incubated with phalloidin-Alexa Fluor 488 (Beyotime, #C2201S) diluted 1:100 or BODIPY 493/503 at 1 μg/ml concentration for 1 h at RT in the dark. The samples were then washed three times with 1× PBS for 10 min each at RT. Finally, the samples were mounted in 80% glycerol (Sangon Biotech, #A100854) containing 5 ng/μl DAPI (Invitrogen, #D-1306). Fluorescent signals were visualized using a Zeiss LSM 800 confocal laser scanning microscope (Carl Zeiss MicroImaging), and the images were analyzed with ZEN 2.3 software (Carl Zeiss MicroImaging). The lipid accumulation in eggs was quantified with the fluorescence intensity of BODIPY staining. The mean intensity value of BODIPY staining per μm^2^ was calculated by Zen 2.3 software. Each egg was treated as an independent biological replicate, with seven independent biological replicates per treatment.

### Western blotting assay

The ovaries were dissected from mated adult female flies at 96 h post-emergence. After being washed three times in 1× PBS and homogenized, the tissues were boiled for 15 min in protein loading buffer. The proteins were separated by sodium dodecyl sulfate-polyacrylamide gel electrophoresis at 80 V for 2 h and transferred to polyvinylidene fluoride membranes at 60 V for 2 h. After blocked with 5% non-fat milk (Sangon Biotech, #A600669) in TBST buffer (50 mM Tris-Cl, pH 7.4, 150 mM NaCl, 0.1% Tween 20) for 1 h at RT, the blots were incubated with mCherry primary antibody (1: 2000 dilution, #ab167453) or α-tubulin primary antibody (1: 500 dilution, Beyotime, #AT819) overnight at 4°C. Following washing three times in TBST buffer, the blots were incubated with HRP-labeled secondary antibody (1:5000 in TBST with 5% non-fat milk) for 1 h at RT. After being washed three times in TBST buffer, the Western blot signals were visualized with ECL reagent.

### Fluorescence microscopy

For live-cell experiments, cells were plated onto a 35-mm glass-bottom dish and transfected with Lipofectamine 3000 (Life Technologies) Transfection Reagent by using the constructs indicated. For imaging, the cell medium was replaced with imaging buffer (25 mM Hepes, 125 mM NaCl, 5 mM KCl, 1.3 mM CaCl_2_, 1.2 mM MgSO_4_.7H_2_O, 0.2% BSA, and 20 mM glucose, pH 7.4). Cells were imaged using either the Novel NSR1000 SIM microscope (Novel Optics) or the FV3000 laser scanning confocal microscope (Olympus), equipped with 100× or 60×1.4 NA oil-immersion objectives. The cells were kept in a thermostat-controlled chamber at 37°C throughout the imaging process. The fluorochromes utilized included Alexa Fluor 488 (BODIPY 493/503), Alexa Fluor 568, EGFP, Texas Red (mCherry and Nile red), and DAPI (Hoechst, #33342).

For live-cell imaging and simultaneous optical stimulation, mCherry-expressing cells were identified and imaged using a 561 nm laser, and the 488 nm laser was used for activation of the optogenetic modules and imaging of the GFP-tagged proteins. Laser powers, measured after the objective, were approximately 5 mW for the 488-nm beam and 5 mW for the 561-nm beam. Following stimulation, only the 561-nm laser was used to facilitate the dissociation of CRY2 and CIBN. The spatial light activation was performed using the FRAP module in an FV3000 laser scanning confocal microscope (Olympus), controlled via the sequence manager function. The experiment employed the “LSM imaging” and “stimulation” functions within the sequence manager. Initially, an image was captured before stimulation. The region of interest (ROI) was then photostimulated using 1% of the 488 nm laser power. Imaging and stimulation occurred every 15 s for a total duration of 40 min.

### Microscopic analysis of LDs

Cells grown on coverslips were treated with 200 μM oleate for 24 h, followed by a 6-h treatment with either regular medium (RM), Hanks' Balanced Salt Solution (HBSS), RM supplemented with 100 nM insulin, 15 μM Nocodazole (NOC), or left untreated. Subsequently, cells were stained with Nile red (1:1,000 dilution), Hoechst 33,342 (1:500 dilution), or optionally by Green Actin Tracking Stain (1:1,000 dilution) for 30 min at 37°C. In some instances, LDs were labeled with 1 μg/ml BODIPY 493/503 (Invitrogen) in PBS for 10 min at 37°C. After three washes with PBS, cells were visualized using a confocal microscope (Olympus FV3000) equipped with a 60× oil immersion objective.

LD size, number, and distance from the nucleus were quantified using ImageJ software (NIH) after thresholding individual frames. Particles were identified using the 'Analyze Particles' function in thresholded single sections with size (μm^2^) settings ranging from 0.1 to 10 and circularity from 0 to 1. The distance of LDs from the nucleus was determined by measuring the distance between the center of the LDs and the center of the nucleus. The trajectories of LDs movements were analyzed using Fiji software with the Trackmate plugin. The displacement and velocity of LDs were measured using the DoG detector and Simple LAP tracker in Fiji. The Track index represents individual LDs identified by Trackmate for color-coded visualization of tracked objects.

### Quantification of LDs distribution and colocalization

To analyze organelle distribution in live cells, double-transfected cells were isolated from the original images and superimposed onto a black background in ImageJ to minimize signal interference from adjacent cells. As depicted in Fig. 1G, the cellular periphery was delineated as the region encompassing 70% of the distance from the cell periphery to the cell center, while the region overlying the nucleus was designated as the cellular perinuclear space. Mean intensity and area within these regions of interest (ROIs) were quantified for each time point. The percentage of peripheral LDs is defined as (the total signal − signal in the perinuclear ROI)/total signal, with the percentage of perinuclear LDs defined as the signal in the perinuclear ROI/total signal.

For the colocalization analysis of CRY2 and CIBN-tagged LDs and motor proteins, images were processed using ImageJ software with the 'JACoP' plugin. This plugin was utilized to compute Pearson's correlation coefficient, ranging from −1 (indicating perfect exclusion) to +1 (indicating perfect correlation) for each time point.

To quantify the relative fluorescence intensity of motor proteins associated with LDs, individual LDs within cells were identified through ImageJ's 'Analyze Particles' function, as described above. The regions of interest (ROIs) corresponding to LDs were recorded in ROI Manager for subsequent analysis. The fluorescence intensity of EGFP-tagged motor proteins within these LDs region was then measured by applying the predefined ROIs. Background fluorescence was subtracted using adjacent cytoplasmic regions to ensure accurate quantification.

### Triglyceride measurement

For measurement of triglycerides in cells, LO2 cells were seeded in six-well dishes one day before transfection and then transfected with indicated plasmids, while simultaneously loaded with 200 μM oleate in the dark. Twelve hours post-transfection, the medium was replaced without fetal bovine serum (FBS) but containing 200 μM OA and 1% bovine serum albumin (BSA). The cells were then subjected to pulsed blue light (488 nm, 2 mW/cm^2^) within an incubator using a custom LED array (19) with a 60-s on/off cycle for 16 h or kept in darkness. The medium was collected (800 μl) and centrifuged at 12,000 × g for 5 min. LO2 cells were lysed in 5% Triton X-100 (120 μl). Triglyceride (TAG) levels were quantified using a Triglyceride Assay Kit from BioAssay Systems (ETGA-200, #75878-176) following the manufacturer's protocol.

### Data analysis

Statistical significance of data was evaluated using Student’s *t* test or one-way analysis of variance (ANOVA) with Tukey’s post hoc test (for multiple datasets). Data were presented as average ± SEM. Statistics and graphing were performed using Prism 8 (GraphPad) or Excel (Microsoft). The confocal images were analyzed by ImageJ-win64 (NIH Image). All images were assembled using Adobe Illustrator software.

## Results

### Optogenetic regulation of lipid droplet distribution in cells

To develop optogenetic tools for controlling the localization of LDs, we utilized the CRY2/CIBN heterodimerization system, which has been successfully applied in previous studies to control other organelle distributions within cells (16). These photosensitive proteins dimerize after blue light stimulation, allowing them to be anchored to the organelle of interest and the motor molecules. Two molecular motors were selected for LD transport: the truncated KIF5A, a kinesin family member that directs anterograde transport (20) and BICDN, the N-terminus of the dynein/dynactin interacting protein BICD, which mediates retrograde transport (21). We fused BICDN or KIF5A to the CIBN domain, which heterodimerizes with the CRY2 domain upon blue light stimulation.

To validate the effectiveness of this light-regulated cargo mobility system, we expressed mCherry-CRY2clust in the cytoplasm. This construct contains a short peptide at the C-terminus of the CRY2PHR domain that promotes protein homo-oligomerization after light stimulation (22). Upon blue light illumination, mCherry-CRY2clust formed clusters within seconds. When associated with KIF5A-EGFP-CIBN and BICDN-EGFP-CIBN, these clusters directed movement toward the cell periphery or perinuclear areas, respectively (Supplemental Fig. S1). This demonstration confirms the functionality of our optogenetic system for dynamically controlling the spatial distribution of cellular cargoes, providing a robust tool for studying organelle transport and positioning in living cells.

To achieve light-regulated control of LD movement, we chose perilipin-2 (PLIN2), a ubiquitously expressed lipid droplet surface protein that serves as a marker for LDs (23). We generated the PLIN2(1–191)-mCherry-CRY2 construct (Fig. 1A), where PLIN2(1–191) contains an 11-mer repeat region predicted to form amphipathic helices (AHs) for efficient LD targeting (24). The specific localization of PLIN2(1–191)-mCherry-CRY2 at LDs was confirmed by colocalization with the neutral lipid dye BODIPY 493/503 (BODIPY) (Supplemental Fig. S2F). We demonstrated that LDs could be transported to the cell periphery following light-induced recruitment of KIF5A, and this light-regulated redistribution of LDs was highly reversible (Fig. 1B, C). In COS-7 cells co-transfected with PLIN2(1–191)-mCherry-CRY2 and KIF5A-EGFP-CIBN, LDs exhibited an even distribution before light stimulation (Fig. 1C). After 50 min of pulsed blue light illumination (5 mW 488 nm laser; 60 s interval), LDs redistributed to the cell periphery, as indicated by white arrows (Fig. 1C, Video S1 and Video S3). In contrast, LD distribution remained largely unchanged in cells transfected with the control construct PLIN2(1–191)-mCherry (Fig. 1D). The colocalization of PLIN2(1–191)-mCherry-CRY2 with KIF5A-EGFP-CIBN occurred within seconds (Fig. 1E and Video S5). The motor proteins, bound to LDs, detached in the dark and LD distribution returned to pre-stimulus levels (Fig. 1F). To quantify LD motility, we delineated 70% of the cell contour as the boundary, with the outer region designated as the cell periphery and the inner region as the perinuclear area (Fig. 1G). Quantitative analysis demonstrated that the percentage of LDs at the cell periphery increased from 20% to approximately 80% following blue light illumination (Fig. 1H). The dynamics of LD movement peaked within the first few minutes and stabilized after about 50 min. Furthermore, the light-induced redistribution of LDs was reversible, with peripheral LDs increasing under blue light and decreasing in darkness (Fig. 1H and Video S1).

Similarly, we fused CIBN with BICDN to harness the dynein-mediated LD movement towards the perinuclear region (Fig. 2A). In live cells, BICDN-EGFP-CIBN and PLIN2(1–191)-mCherry-CRY2 bound together after 60 s of blue light stimulation (Fig. 2B, C and Video S6). Live-cell imaging of COS-7 cells transfected with BICDN-EGFP-CIBN and PLIN2(1–191)-mCherry-CRY2 showed that LDs repositioned to the perinuclear area after 30 min of pulsed blue light illumination (5 mW 488 nm laser; 60 s interval), with the proportion in the perinuclear region increasing from 50% to 90% (Fig. 2D, F and Video S2). Three-dimensional confocal imaging confirmed that the majority of LDs were redistributed to the perinuclear area after light stimulation (Video S4). The light-induced perinuclear redistribution of LDs was also reversible, through light-regulated motor localization on LDs (Fig. 2E, F and Video S2).

Additional experiments confirmed the potency and specificity of Opto-LDs in regulating the intracellular localization of LDs. Overexpression of these two motor proteins alone did not affect LD distribution, nor did it alter their number, size or microtubule structure (Supplemental Fig. S2A-E). LDs exhibited highly dynamic behavior in the basal state, with rapid, increased long-range directional movements only observed following blue light stimulation (Supplemental Fig. S2G-J). Indeed, live cell imaging revealed that most LDs bound to microtubules upon light stimulation and were transported along them, as shown by the colocalization of the fluorescent protein-tagged microtubule-binding domain of ensconsin (EMTB) with LDs (Fig. 2G, Supplemental Fig. S3A and Videos S7–S9). Notably, the endogenous microtubule network displayed a complex architecture, distinct from the simplified radial distribution often depicted in schematic models (Figs. 1B and 2A). Consistent with established principles, microtubule plus-ends were predominantly localized near the cell periphery, while minus-ends were concentrated at the microtubule-organizing center (MTOC) adjacent to the nucleus. Based on this spatial organization, we quantified polar transport by measuring the distance between LDs and the nucleus: perinuclear LDs were classified as negative transport, whereas pericellular LDs were classified as positive transport. Importantly, this metric reflects changes in distribution rather than polar transport efficiency. Additionally, we demonstrated that by integration with the FRAP module, the Opto-LDs allow for spatial regulation of LD transport in single cells (Supplemental Fig. S3B).

### Light-induced relocation of LDs modulates lipid metabolism in hepatocytes

LDs play a crucial role in maintaining lipid homeostasis in hepatocytes and are implicated in a variety of physiological and pathological processes. The intracellular transport of LDs in hepatocytes is intricately linked to lipid metabolism. In a fed state, insulin signaling cooperated with kinesin motors to transport LDs to the cell peripheral for very-low-density lipoprotein (VLDL) production (25). Conversely, in a fasted state, AMPK promotes LD dispersion (26, 27), which is also associated with mitochondria-LD interactions in the liver (28). Consistent with these findings, we observed distinct LD distribution patterns in LO2 hepatocytes under different metabolic conditions (Supplemental Fig. S4A). The cells were loaded with oleic acid (OA) and treated with Hanks' Balanced Salt Solution (HBSS) or regular medium containing 100 nM insulin to simulate the fasted and fed conditions, respectively. Insulin treatment markedly increased the proportion of peripheral LDs, while nutrient deprivation induced by HBSS incubation led to a decrease in peripheral LDs (Supplemental Fig. S4B). Additionally, cellular triglyceride (TG) levels and secretion varied under these three conditions (Supplemental Fig. S4C, D). To demonstrate that the dynamic distribution of LDs relies on an intact microtubule system, we treated LO2 hepatocytes with nocodazole (15 μM for 6 h), which significantly reduced the presence of LDs at the cell periphery (Supplemental Fig. S4E, F).

We first demonstrated that the Opto-LDs system can effectively and rapidly modulate LD distribution in LO2 hepatocytes (Fig. 3A and Supplemental Fig. S4G, H). To determine whether this light-induced LD redistribution directly influences hepatic lipid metabolism, we subsequently examined very-low-density lipoprotein (VLDL) secretion. LDs serve as a source of lipids for VLDL assembly and provide a platform for VLDL synthesis. By applying the Opto-LDs to induce peripheral localization of LDs, we observed a substantial increase in triglyceride (TG) secretion in LO2 hepatocytes expressing PLIN2(1–191)-mCherry-CRY2 and KIF5A-EGFP-CIBN, compared to cells kept in the dark or control cells expressing PLIN2(1–191)-mCherry and KIF5A-EGFP-CIBN (Fig. 3B, C). In contrast, the perinuclear accumulation of LDs induced by light resulted in a marked decrease in VLDL secretion (Fig. 3D, E). This suggests that the peripheral accumulation of LDs enhances lipid mobilization and VLDL secretion in hepatocytes (Fig. 3F), potentially due to increased interactions between the endoplasmic reticulum (ER) and LDs (29). Together, these results, consistent with previous studies (25, 30), suggest a direct link between the spatial distribution of LDs and lipid metabolism in hepatocytes.

### Sustained light-induced relocation of LDs in *Drosophila* oocyte

LDs in the early *Drosophila* embryo are formed during oogenesis in nurse cells and are transferred into the developing oocyte through the ring canals (Fig. 4A). These LDs are believed to be the primary energy source for the embryo, with a substantial portion of the stored lipids being consumed throughout embryogenesis (31). In *Drosophila* oocytes and embryos, LDs exhibit high motility, which is tightly regulated (32). To demonstrate the principle of mobilizing LDs in living animals during development, we chose to control LD movement during oogenesis and examined the biological consequences of these manipulations.

We utilized a germline-specific Gal4 driver (nanos-Gal4) to express the Opto-LDs system post-oocyte specification, enabling light-regulated control of LD movement. Lsd-1, an ortholog of human perilipin 1 (33), was employed for LD targeting in *Drosophila*. We generated bicistronic vectors containing P2A to allow for co-expression of Opto-LDs constructs, specifically Lsd-1-mCherry-CRY2-P2A-KIF5A-CIBN and the control construct Lsd-1-mCherry-P2A-KIF5A-CIBN, which lacks CRY2. Both Lsd-1-mCherry and Lsd-1-mCherry-CRY2 labeled LDs in fly ovaries under both dark and light conditions, as indicated by BODIPY staining (Supplemental Fig. S5A). Female flies equipped with these optogenetic tools were mated with wild-type *w1118* male flies, subjected to either dark or light conditions, and analyzed for biological outcomes (Fig. 4B).

Confocal imaging of the ovarioles in adult ovaries revealed that LDs distribution in the Opto-LDs expressing group was significantly altered after blue light illumination, in contrast to the uniformly distributed LDs in the control group (Fig. 4C and Supplemental Fig. S5A). Upon light stimulation, the expression of Opto-LDs induced clustering of LDs in both the interconnected nurse cells and the oocyte after the middle stage of oogenesis, a period when nurse cells begin generating substantial amounts of LDs. Lipid accumulation in ovaries decreased, as shown by the reduction of the BODIPY staining (Supplemental Fig. S5A, B). By tallying the tubes with eggs for each pair of crossed flies, we found that the redistribution of LDs did not affect ovulation capacity (Supplemental Fig. S5D), a finding further supported by our egg-laying experiments (Fig. 4D). However, light-induced relocation of LDs led to a marked decrease in female fertility, as indicated by the reduced emergence of adult flies for the Opto-LDs expressing group (Supplemental Fig. S5E). We analyzed the hatching rates of the eggs with control or Opto-LDs expression under dark or light conditions and found a significant reduction in the Opto-LDs-expressing group upon blue light stimulation (Fig. 4E). The expression levels of Lsd-1-mCherry or Lsd-1-mCherry-CRY2 remained unchanged before and after light stimulation (Supplemental Fig. S5F, G). However, Lsd-1-mCherry appeared to have a higher expression level than the Lsd-1-mCherry-CRY2 in ovaries, as indicated by Western blot analysis with an anti-mCherry antibody. This difference may explain the lower hatching rate in the control group under both light and dark conditions (Fig. 4E).

## Discussion

In this study, we developed an Opto-LDs module for the precise control of intracellular LDs translocation. The light-induced movement of LDs is reversible, repeatable, and can be reorganized to subcellular regions within minutes (Figs. 1C and 2D). Unlike chemical and genetic methods, which may result in irreversible phenotypes or induce cytotoxicity and signaling cross-talk due to off-target effects or pleiotropy, our approach offers a non-invasive alternative. For instance, genetic ablation of kinesin-1, commonly used to study LD transport in embryos, is problematic because kinesin-1 has multiple functions. Embryos lacking kinesin-1 fail to cellularize and perish during mid-Phase II, potentially obscuring the movements of LDs (31, 34). However, the Opto-LDs also have their drawbacks; overexpression of motors might influence the transport of other cellular cargoes, and the weak self-aggregation effect of CRY2 may lead to self-aggregation of anchored LDs. In the ovaries expressing Opto-LDs, mild LD clustering was observed under dark conditions (Fig. 4C and Supplemental Fig. S5A), likely due to CRY2’s self-aggregation effects. The normal fertility and egg hatching rates (Fig. 4D, E) suggested that this mild clustering did not have significant physiological effects. Our study showed that the peripheral relocation of LDs in hepatocytes promotes VLDL secretion (Fig. 3C), indicating that LD localization is associated with lipid metabolism. This effect is likely due to increased interaction between LDs and the smooth endoplasmic reticulum (sER), which elevates lipoprotein synthesis and the secretion of mature VLDL (29). In the fed state, insulin signaling facilitates the peripheral transport of LDs by recruiting kinesin-1 motors, which promotes contact with the sER for VLDL secretion (25, 30). LDs play multifunctional roles, with motility being crucial for lipid metabolism. This includes regulating LD outgrowth from the ER (35), transferring fatty acids to mitochondria (36) and controlling the degradation of LDs by autophagic lysosomes (37). Typically, hepatic lipid homeostasis is maintained through increased lipoprotein secretion. However, in non-alcoholic fatty liver disease (NAFLD), lipoprotein secretion is insufficiently enhanced (38). Our findings suggest a reciprocal relationship between the dynamic motility of LDs and lipid metabolism, with nutritional states influencing LD distribution and, in turn, affecting metabolism in hepatocytes.

During *Drosophila* oogenesis, altering the standard distribution of LDs with light significantly impairs the hatching of the embryos (Fig. 4E). Notably, the ovulation capacity remains unaffected (Fig. 4D and Supplemental Fig. S5D). In contrast, inhibiting the transport of cytoplasmic materials, including LDs, through the ring canal by knockdown of Short stop (Shot), a *Drosophila* spectraplakin, halts oocyte growth, resulting in a distinctive small oocyte phenotype (7). The movement of LDs has been linked to nutrient transport, biogenesis and breakdown of lipid droplets, protein and lipid exchange between cellular compartments, and even the maturation of viruses (39). Additionally, LDs serve as protein carriers enabling embryos to accumulate extranuclear histone stores and supply histones for chromatin assembly during periods of heightened demand (40). Our results suggest that the proper distribution of LDs plays a crucial role in embryonic development. However, further study is required to elucidate how the distribution and motility of LDs affect embryogenesis in flies. Consequently, our findings, alongside other research, underscore the importance of effective LD motility and targeted transport for the regulation of cellular processes. It must be acknowledged that the Opto-LDs system has several limitations. For instance, controlling the specific stage of *Drosophila* oogenesis in vivo is challenging, complicating the interpretation of the relationship between failed embryo development and aberrant LD distribution. Additionally, since microtubule organization in these tissues remains uncharacterized, the mechanism underlying light-induced LD clustering in *Drosophila* remains unclear. Two plausible explanations may account for this observation: excessive kinesin accumulation on LDs or the relatively weak self-aggregation propensity of CRY2. Furthermore, demonstrating the reversibility of the tool in vivo is problematic due to the continuous nature of oogenesis.

In summary, our study presents an optogenetic approach to manipulating LD movement in living cells and *Drosophila* embryos, achieving reversibility and precise spatiotemporal control in cultured cells. These investigations are poised to provide insights into the intrinsic characteristics of LDs and to elucidate their critical role in organismal physiology and development.

## Data availability

All data supporting the findings of this study are available either in the article and/or in its supplementary information files. Other source data are provided upon request.

## Supplemental data

This article contains supplemental data.

## Conflict of interest

The authors declare that they have no conflicts of interest with the contents of this article.

## Supplementary Data

Supplementary Figures Supplementary Figure S1: Light-induced formation of CRY2 clusters and their relocation to the cell periphery or perinuclear areas. A-C. Representative confocal images demonstrate the reversibility of the formation of CRY2 clusters and their translocate to the cell periphery in a COS-7 cell transfected with mCherry-CRY2clust and KIF5A-EGFP-CIBN under pulsed blue light illumination (5 mW 488 nm laser; 20 s interval) for 30 min (A). The cell boundary and nucleus were marked in (B). Representative time-lapse images illustrate the blue light-induced CRY2 clusters translocate to the cell periphery (C). Enlarged views of the regions enclosed by the white box in A are displayed. White arrows indicate the same cluster movements in different frames. D-F. Representative confocal images demonstrate the reversibility of the formation of CRY2 clusters and their translocate to the perinuclear area in a COS-7 cell expressing PLIN2(1–191)-mCherry-CRY2 and BICDN-EGFP-CIBN under pulsed blue light illumination (5 mW 488 nm laser; 20 s interval) for 30 min (D). The cell boundary and nucleus were marked in (E). Time-lapse images show the light-induced CRY2 clusters moving toward the cell perinuclear area (F). Enlarged views of the regions enclosed by the white box in D are displayed. White arrows indicate the same cluster movements in different frames. Scale bars: 20 μm (A and D), 5 μm (C and F). Supplementary Figure S2: Overexpression of Opto-LDs does not alter the localization, number, size or dynamics of intracellular LDs. A. The confocal imaging of COS-7 cells (WT), COS-7 cells transfected with KIF5A-EGFP-CIBN (KIF5A) or BICDN-EGFP-CIBN (BICDN) stained with BODIPY558/568 C12. B-D. Quantifications of LD distribution (B), number (C), and size (D) in A (n = 15). E. Live-cell imaging of COS-7 cells expressing EMTB-miRFP670nano3 with or without KIF5A-EGFP-CIBN (KIF5A) or BICDN-EGFP-CIBN (BICDN). F. Co-localization of PLIN2(1–191)-mCherry-CRY2 (red) with BODIPY 493/503 (green) in COS-7 cells. G. COS-7 cells transfected with PLIN2(1–191)-mCherry-CRY2 were illuminated by blue light (488 nm) for 30 min. The trajectories of LDs are shown. Color map means displacement, 0–10 μm. H. COS-7 cells co-transfected with PLIN2(1–191)-mCherry-CRY2 and KIF5A-EGFP-CIBN were illuminated by blue light (488 nm) for 30 min. The trajectories of LDs are shown. Color map means displacement, 0–10 μm. I-J. The track displacement of LDs in G and H respectively. The mean displacements were marked, and the Track index means individual LDs identified by Trackmate. Data are represented as mean ± SEM. ∗P < 0.05; ∗∗P < 0.01; ∗∗∗P < 0.001 by *t* test. Scale bars: 20 μm (A and E), 10 μm (F-H). Supplementary Figure S3: Spatiotemporal perturbation of LD transport with light. A. Confocal microscopy analysis of COS-7 cells expressing EMTB-miRFP647nano3 (microtubule marker) and PLIN2(1–191)-mCherry-CRY2 under blue light stimulation conditions. Cells were co-transfected with either: (1) no motor construct (Control), (2) KIF5A-EGFP-CIBN (peripheral LD enrichment), or (3) BICDN-EGFP-CIBN (perinuclear LD enrichment). Scale bars: 10 μm. B. COS-7 cells were co-transfected with PLIN2(1–191)-mCherry-CRY2 (red) and either KIF5A-EGFP-CIBN (top panel), or BICDN-EGFP-CIBN (bottom panel). The indicated region (blue circle) at the center of the stimulation cell (dotted circle) was locally illuminated with a 488 nm laser. The whole cell distributions of PLIN2(1–191)-mCherry-CRY2-labeled LDs before and after light stimulation were shown. Scale bar: 40 μm. Supplementary Figure S4: Both metabolic conditions and light perturbation induce alterations in LD distribution within hepatocytes. A. Live-cell confocal images of LO2 cells incubated with oleic acid (OA) for 24 h, followed by treatment with regular medium (RM), Hanks' Balanced Salt Solution (HBSS), or RM supplemented with 100 nM insulin for 6 h. Cells were fixed and stained with Nile Red (LDs, red), Hoechst 33342 (nucleus, blue), and phalloidin-Alexa Fluor 488 (F-actin, green). B. Quantification of the average distance between LDs and the nucleus from images in (A) (n = 26 cells analyzed per condition). C-D. Triglyceride (TG) levels in LO2 cells: intracellular TG levels (C) and secreted TG levels (D) were measured after cells were maintained in regular medium (RM) or medium supplemented with OA for 24 h, followed by incubation with HBSS or 100 nM insulin for an additional 6 h. E. Live-cell confocal imaging of LO2 cells treated with OA and either exposed to 15 mM nocodazole (NOC) or left untreated for 6 h. Cells were stained with Nile Red (LDs, red) and Hoechst 33342 (nucleus, blue). F. Quantification of the average distance between LDs and the nucleus from images in (D) (n = 10 cells analyzed per condition). G. Live-cell imaging and colocalization analysis of cells co-expressing PLIN2(1–191)-mCherry-CRY2 and KIF5A-EGFP-CIBN before and after blue light stimulation (488 nm, 6 min) in low-resolution imaging. Scale bars: 10 μm. Imaging parameters: 512 × 512 pixels. H. Live-cell imaging and colocalization analysis of cells co-expressing PLIN2(1–191)-mCherry-CRY2 and BICDN-EGFP-CIBN before and after blue light stimulation (488 nm, 6 min) in low-resolution imaging. Scale bars: 10 μm. Imaging parameters: 512 × 512 pixels. Supplementary Figure S5: Sustained light-induced relocation of LDs in *Drosophila* oocyte. A. Representative confocal images of ovarioles from *nanosGal4> Lsd-1-mCherry*^*P2A*^*-KIF5A-CIBN* (Control) and *nanos-Gal4> Lsd-1-mCherry-CRY2*^*P2A*^*-KIF5A-CIBN* (Opto-LDs) flies in the dark and after light stimulation. Ovaries were stained with BODIPY 493/503 and DAPI, the bottom panel illustrating each channel. The mCherry indicates Lsd-1-labeled LDs. Scale bar 20 μm. B. The lipid accumulation in eggs was quantified by the intensity of BODIPY staining (n = 6). C. Representative confocal images of the early follicles from the wild type *w118* and *nanosGal4> Lsd-1-mCherry*^*P2A*^*-KIF5A-CIBN.* Ovaries were stained with BODIPY 493/503 and DAPI. D-E. The proportion of tubes with eggs (D) and adult flies (E) resulting from crosses of 100 pairs of female and male with indicated genotypes. Single female and male were cohabited in a same tube for more than 7 days before they were transferred to a fresh tube. Eggs were examined after 2 days. The emerged flies were counted 14 days later. All the experiments were performed under either dark or blue light illuminated conditions as indicated. F. Western blot analysis of mCherry-tagged protein levels in Control and Opto-LDs ovaries in the dark and after blue light stimulation. G. Quantification of ratios of mCherry: Tublin using immunoblots shown in (F) (n = 6).

Video S1 **Video S1:** Optogenetic control of LD movement to the cell periphery. Time-lapse confocal images showing a COS-7 cell transfected with PLIN2(1–191)-mCherry-CRY2 and KIF5A-EGFP-CIBN underwent two cycles of blue light stimulation. The cell was stimulated with 488 nm laser for 50 min, followed by 80 min of recovery in the dark, and stimulated with blue light for another 50 min. Stimulation parameters: 5 mW 488 nm laser; 60-s interval. Imaging parameters in the dark: only 561 nm laser; 120-s interval. Time: 00:00 indicates hour: minutes. The video was compressed at 10 frames per second for demonstration. Note: The video illustrates that the translocation of LD toward the cell periphery in response to light illumination is reversible and repeatable. Scale bar: 10 μm.

Video S2 **Video S2:** Optogenetic control of LD movement to cell perinuclear area. Time-lapse confocal images showing a COS-7 cell transfected with PLIN2(1–191)-mCherry-CRY2 and BICDN-EGFP-CIBN underwent two cycles of blue light stimulation. The cell was stimulated with a 488 nm laser for 35 min, followed by an 80-min recovery period in the dark, and stimulated with blue light for another 45 min. Stimulation parameters: 5 mW 488 nm laser; 60-s interval. Imaging parameters in the dark: only 561 nm laser; 120-s interval. Time: 00:00 indicates hour: minutes. The video was compressed at 10 frames per second for demonstration. Note: The video illustrates that the translocation of LDs toward the cell perinuclear area in response to light illumination is reversible and repeatable. Scale bar: 10 μm.

Video S3 **Video S3:** Three-dimensional reconstruction images showing the redistribution of LDs in a COS-7 cell expressing PLIN2(1–191)-mCherry-CRY2 and KIF5A-EGFP-CIBN to the perinuclear area before and after blue light stimulation. The cell was imaged with confocal microscope and was stimulated with 5 mW 488 nm laser at 60-s interval for 30 min. Scale bar: 10 μm.

Video S4 **Video S4:** Three-dimensional reconstruction images showing the redistribution of LDs in a COS-7 cell expressing PLIN2(1–191)-mCherry-CRY2 and BICDN-CIBN to the perinuclear area before and after blue light stimulation. The cell was imaged with confocal microscope and was stimulated with 5 mW 488 nm laser at 60-s interval for 30 min. Scale bar: 10 μm.

Video S5 **Video S5:** Real-time visualization of peripheral LD enrichment reveals kinesin motor binding dynamics. Time-lapse confocal microscopy of a LO2 hepatocyte co-expressing PLIN2(1–191)-mCherry-CRY2 and KIF5A-EGFP-CIBN following blue light stimulation (488 nm laser, 20-s intervals for 6 min). Image acquisition parameters: 512 × 512 pixels resolution. The video was compressed at 10 frames per second for demonstration. Note: The sequence demonstrates the light-dependent recruitment of KIF5A motors to LDs. Scale bar: 10 μm.

Video S6 **Video S6:** Real-time visualization of perinuclear LD enrichment reveals dynein motor binding dynamics. Time-lapse confocal microscopy of a LO2 hepatocyte co-expressing PLIN2(1–191)-mCherry-CRY2 and BICDN-EGFP-CIBN following blue light stimulation (488 nm laser, 20-s intervals for 6 min). Image acquisition parameters: 512 × 512 pixels resolution. The video was compressed at 10 frames per second for demonstration. Note: The sequence demonstrates the light-dependent recruitment of BICDN motors to LDs. Scale bar: 10 μm.

Video S7 **Video S7-9:** Microtubule dynamics during light-induced LD transport. Time-lapse confocal microscopy of COS-7 cells expressing EMTB-miRFP647nano3 (microtubule marker) and PLIN2(1–191)-mCherry-CRY2 under blue light stimulation for 30 min. Cells were co-transfected with either: no motor construct (Control) (Video S7), KIF5A-EGFP-CIBN (peripheral LD enrichment) (Video S8), or BICDN-EGFP-CIBN (perinuclear LD enrichment) (Video S9). Time: 00:00 indicates hour: minutes. The video was compressed at 10 frames per second for demonstration. Note: The microtubule network remains largely unaltered following expression of motor constructs and LD-targeted proteins under all experimental conditions. Scale bar: 10 μm.
