# Supplementary material for: Optogenetic perturbation of lipid droplet localization affects lipid metabolism and development in Drosophila
Source: J Lipid Res. 2025 Jun 20;66(8):100848. doi: 10.1016/j.jlr.2025.100848 (PMC12302289; doi:10.1016/j.jlr.2025.100848)

Supplementary Figure S1

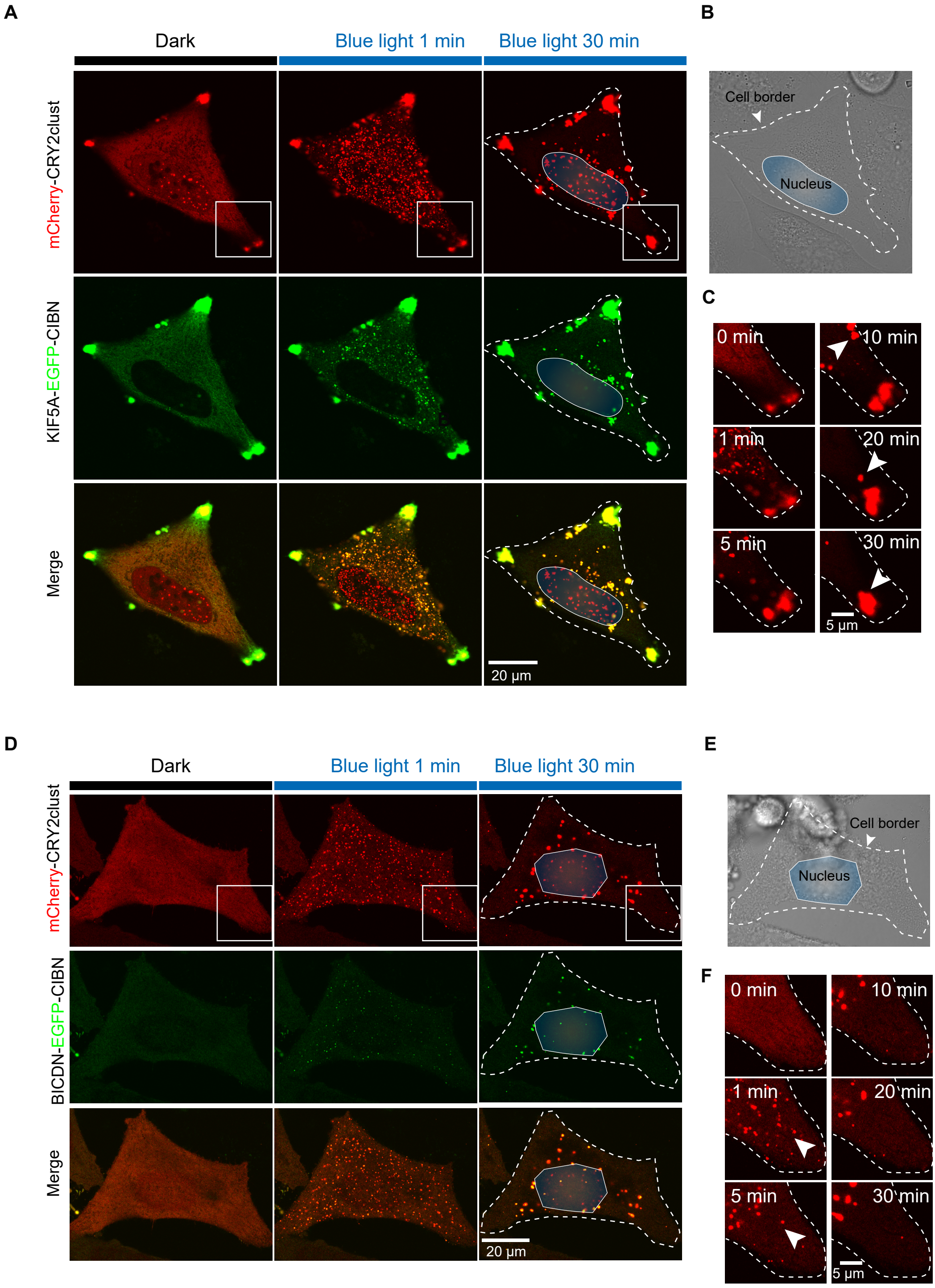

Supplementary Figure S2

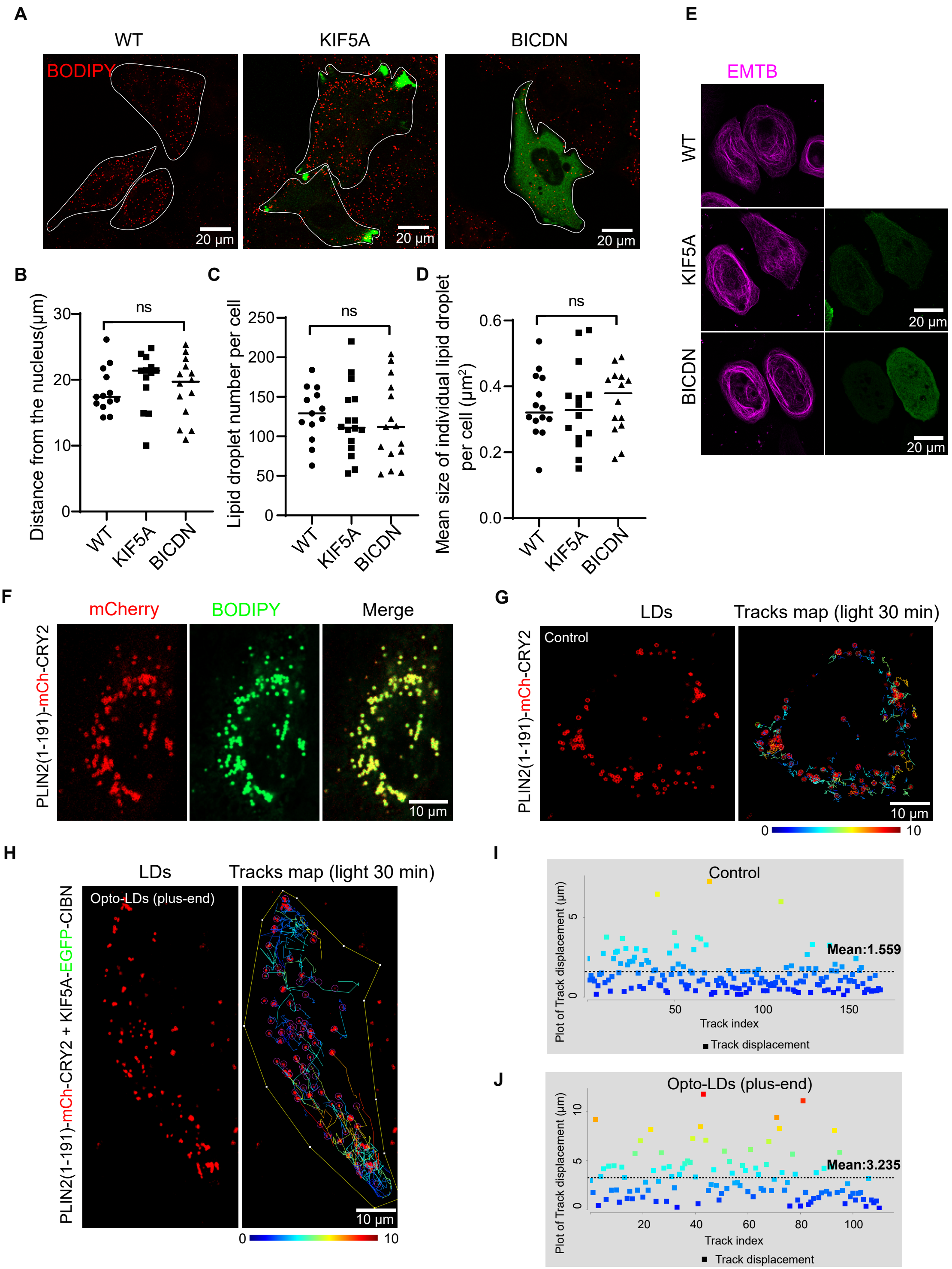

Supplementary Figure S3

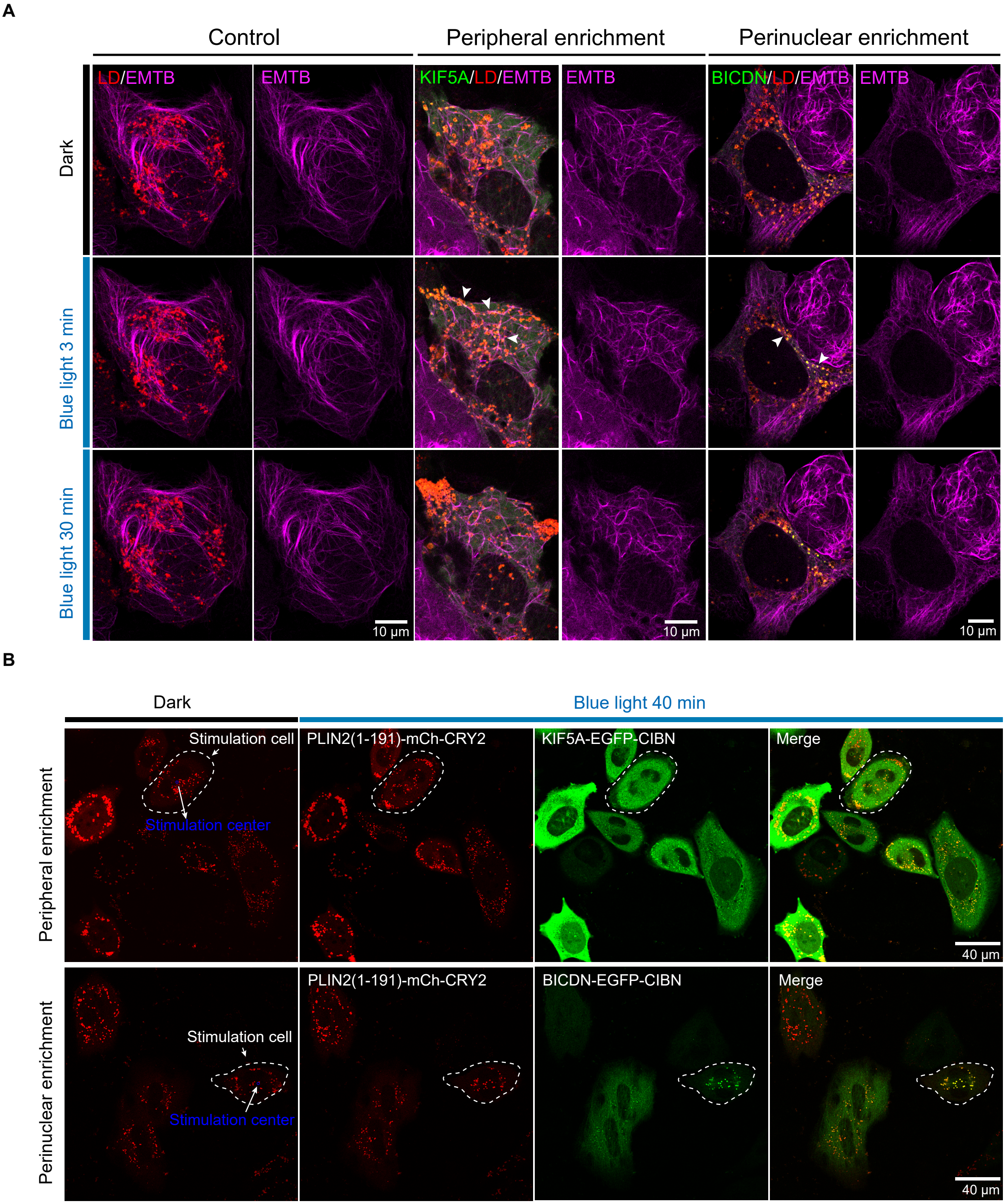

Supplementary Figure S4

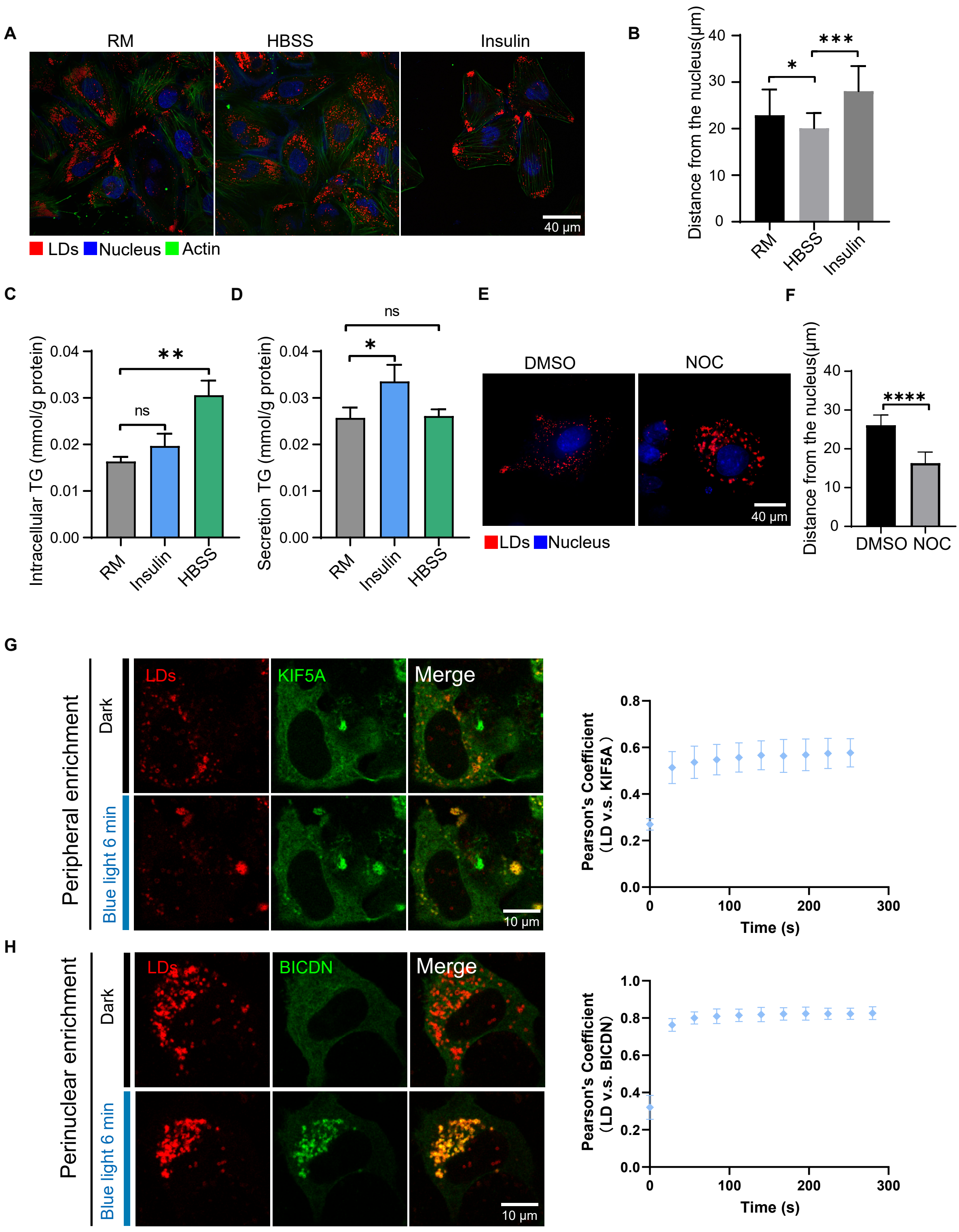

Supplementary Figure S5

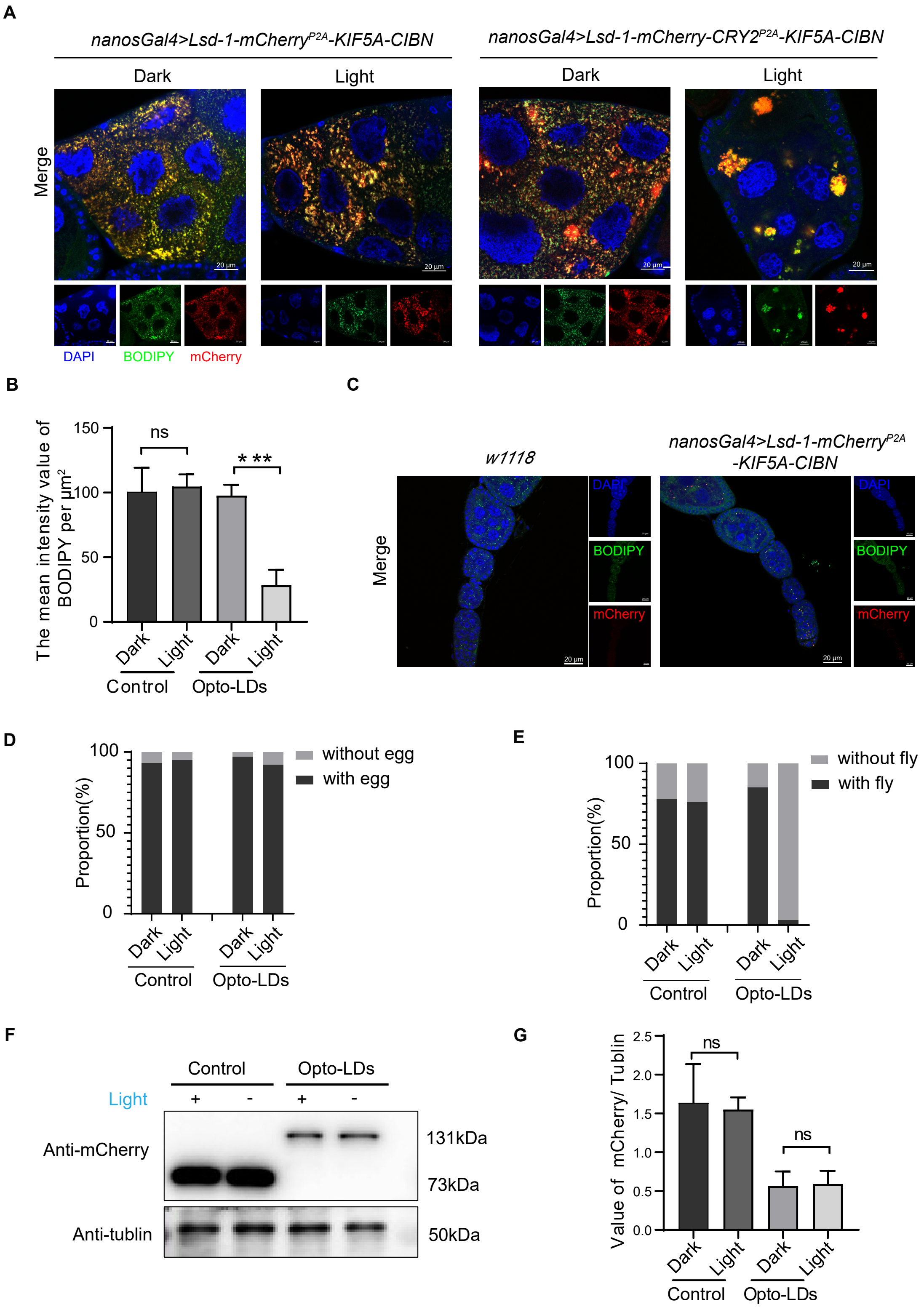

Supplement: Supplementary Figures — Supplementary Figure S1: Light-induced formation of CRY2 clusters and their relocation to the cell periphery or perinuclear areas. A-C. Representative confocal images demonstrate the reversibility of the formation of CRY2 clusters and their translocate to the cell periphery in a COS-7 cell transfected with mCherry-CRY2clust and KIF5A-EGFP-CIBN under pulsed blue light illumination (5 mW 488 nm laser; 20 s interval) for 30 min (A). The cell boundary and nucleus were marked in (B). Representative time-lapse images illustrate the blue light-induced CRY2 clusters translocate to the cell periphery (C). Enlarged views of the regions enclosed by the white box in A are displayed. White arrows indicate the same cluster movements in different frames. D-F. Representative confocal images demonstrate the reversibility of the formation of CRY2 clusters and their translocate to the perinuclear area in a COS-7 cell expressing PLIN2(1–191)-mCherry-CRY2 and BICDN-EGFP-CIBN under pulsed blue light illumination (5 mW 488 nm laser; 20 s interval) for 30 min (D). The cell boundary and nucleus were marked in (E). Time-lapse images show the light-induced CRY2 clusters moving toward the cell perinuclear area (F). Enlarged views of the regions enclosed by the white box in D are displayed. White arrows indicate the same cluster movements in different frames. Scale bars: 20 μm (A and D), 5 μm (C and F). Supplementary Figure S2: Overexpression of Opto-LDs does not alter the localization, number, size or dynamics of intracellular LDs. A. The confocal imaging of COS-7 cells (WT), COS-7 cells transfected with KIF5A-EGFP-CIBN (KIF5A) or BICDN-EGFP-CIBN (BICDN) stained with BODIPY558/568 C12. B-D. Quantifications of LD distribution (B), number (C), and size (D) in A (n = 15). E. Live-cell imaging of COS-7 cells expressing EMTB-miRFP670nano3 with or without KIF5A-EGFP-CIBN (KIF5A) or BICDN-EGFP-CIBN (BICDN). F. Co-localization of PLIN2(1–191)-mCherry-CRY2 (red) with BODIPY 493/503 (green) in COS-7 cel [file mmc1.pdf]
